# *Delirium* in a Latin American intensive care unit. A prospective cohort study of mechanically ventilated patients

**DOI:** 10.5935/0103-507X.20170058

**Published:** 2017

**Authors:** Patricia Mesa, Ignacio José Previgliano, Sonia Altez, Silvina Favretto, María Orellano, Cinthya Lecor, Ana Soca, Ely Wesley

**Affiliations:** 1 Intensive Care Unit, Hospital Pasteur - Montevideo, Uruguay.; 2 Intensive Care Department, Hospital Juan A. Fernández, Maimonides University - Buenos Aires, Argentina.; 3 British Hospital - Montevideo, Uruguay.; 4 Veteran’s Affairs Geriatric Research Education and Clinical Center of the Tennessee Valley - Nashville, TN, USA.; 5 Vanderbilt University Medical Center - Nashville, TN, USA.

**Keywords:** Delirium, Respiration, artificial, Mortality, Brain organ dysfunction, Analgesia, Concencious sedation

## Abstract

**Objective:**

To establish the prevalence of *delirium* in a general intensive care unit and to identify associated factors, clinical expression and the influence on outcomes.

**Methods:**

This was a prospective cohort study in a medical surgical intensive care unit. The Richmond Agitation-Sedation Scale and Confusion Assessment Method for the Intensive Care Unit were used daily to identify *delirium* in mechanically ventilated patients.

**Results:**

In this series, *delirium* prevalence was 80% (N = 184 delirious patients out of 230 patients). The number of patients according to *delirium* psychomotor subtypes was as follows: 11 hyperactive patients (6%), 9 hypoactive patients (5%) and 160 mixed patients (89%). Multiple logistic regression modeling using *delirium* as the dependent outcome variable (to study the risk factors for *delirium*) revealed that age > 65 years, history of alcohol consumption, and number of mechanical ventilation days were independent variables associated with the development of *delirium*. The multiple logistic regression model using hospital mortality as the dependent outcome variable (to study the risk factors for death) showed that severity of illness, according to the Acute Physiology and Chronic Health Evaluation II, mechanical ventilation for more than 7 days, and sedation days were all independent predictors for excess hospital mortality.

**Conclusion:**

This Latin American prospective cohort investigation confirmed specific factors important for the development of *delirium* and the outcome of death among general intensive care unit patients. In both analyses, we found that the duration of mechanical ventilation was a predictor of untoward outcomes.

## Abstract (pt)

**Objetivo:**

Estabelecer a prevalência do *delirium* em uma unidade de terapia intensiva geral e identificar os fatores associados, sua expressão clínica e sua influência no desfecho.

**Métodos:**

Trata-se de um estudo prospectivo de coorte em uma unidade de terapia intensiva clínico-cirúrgica. Avaliamos os pacientes diariamente, com a *Richmond Agitation-Sedation Scale* e a *Confusion Assessment Method for the Intensive Care Unit*, com o objetivo de identificar *delirium* nos pacientes mecanicamente ventilados.

**Resultados:**

Nesta série de casos, a prevalência de *delirium* foi de 184 pacientes com *delirium* em um total de 230 pacientes. O subtipo de *delirium* psicomotor foi hiperativo em 11 pacientes (6%), hipoativo em 9 (5%) e misto em 160 (89%). Uma modelagem de regressão logística múltipla, com *delirium* como a variável de desfecho dependente (para avaliar os fatores de risco para *delirium*), revelou que idade acima de 65 anos, histórico de consumo de álcool e dias em uso de ventilação mecânica foram variáveis que se associaram independentemente com o desenvolvimento de *delirium*. Um modelo de regressão logística múltipla, que utilizou mortalidade hospitalar como variável de desfecho dependente (para estudar os fatores de risco para óbito), mostrou que o índice de severidade da doença, como o aferido segundo o escore *Acute Physiology and Chronic Health Evaluation II*, o uso de ventilação mecânica por mais de 7 dias e o número de dias de sedação foram as dependentes preditoras de mortalidade hospitalar mais elevada.

**Conclusão:**

Este estudo latino-americano de coorte confirmou os fatores específicos importantes para *delirium* e o desfecho óbito entre pacientes admitidos a uma unidade de terapia intensiva geral. Em ambas as análises, identificamos que a duração da ventilação mecânica é um preditor de desfechos desfavoráveis.

## INTRODUCTION

*Delirium* is a form of brain-organ dysfunction associated with significantly higher mortality, longer times on mechanical ventilation, extended time in the intensive care unit (ICU) and increased hospital length of stay (LOS) as well as cognitive impairments at one year after hospital discharge.^(1-3)^ Surveys in Latin American,^(4)^ Brazil^(5)^ and Uruguay^(6)^ revealed that most of the surveyed intensivists did not use a tool for *delirium* evaluation in their practices.

However, the Society of Critical Care Medicine (SCCM) is conducting the ICU Liberation Collaborative and the ABCDEFCare Bundle to implement the Pain, Agitation, *and Delirium* Guidelines (www.icudelirium.org and www.iculiberation.org).

These mechanisms of quality improvement can be adapted for use in Latin American countries; however, prospective data are required to understand the epidemiology of this area of ICU care. We also collected outcomes and a myriad of clinical data at baseline to conduct statistical modeling and analyze the following two main questions: What are the risk factors for *delirium*? What are the risk factors for hospital mortality?

The aim of this study was to establish the prevalence of delirium in a general ICU in Uruguay and to identify predictors of *delirium* and mortality.

## METHODS

This study was conducted between April 20, 2014 and April 20, 2015. The setting was a 25-bed general ICU of the *Hospital Pasteur,* in Montevideo, Uruguay. A general ICU accepts medical, coronary, surgical and trauma patients.

*Hospital Pasteur* is a non-university facility, although it has a university medical residencies program. This study design was a single-center prospective cohort study. The inclusion criteria were patients admitted to the ICU and were 18 years of age and older who required mechanical ventilation (MV) for more than 48 hours. The exclusion criteria were as follows: use of noninvasive mechanical ventilation, patients transferred from another medical facility, patients being transferred to another medical facility that prevented adequate follow-up, ICU readmission in less than 48 hours after discharge and patients with severe neurological and neuropsychiatric pathology.

Patients with severe neurological and neuropsychiatric pathology were defined as those with a disability that makes communication impossible between patients and the health team; therefore, questions would have to be formulated using the Confusion Assessment Method for the Intensive Care Unit (CAM-ICU).

The *Hospital Pasteur*, State Health Services Administration (ASSE), Montevideo, Uruguay's Research Ethics Committee approved the study (Acta 29-006-3-650-2016), and informed consent was exempted.

### Education and training in *delirium* monitoring instruments

From January 1^st^ to March 31^st^, 2014, training workshops for the Richmond Agitation-Sedation Scale (RASS)^(7,8)^ and the CAM-ICU^(9-11)^ evaluation were conducted during every shift for doctors, residents, and nurses.

### *Delirium* definition and diagnosis

We used the Diagnostic and Statistical Manual of Mental Disorders 5^th^ edition (DSM-V)^(12)^ and a validated Spanish version of the CAM-ICU^(10,11,13)^ as diagnostic tools to follow a prospective cohort of ICU patients. A *delirium* diagnosis was determined when the CAM-ICU was positive.

We defined *delirium* as a disturbance or fluctuation in mental status from baseline accompanied by inattention and either altered level of consciousness or disorganized thinking.^(11,12)^

There must be evidence from the history, physical examination, or laboratory findings that the disturbance is a physiologic consequence of another medical condition, substance intoxication or withdrawal.

**Figure 1:**
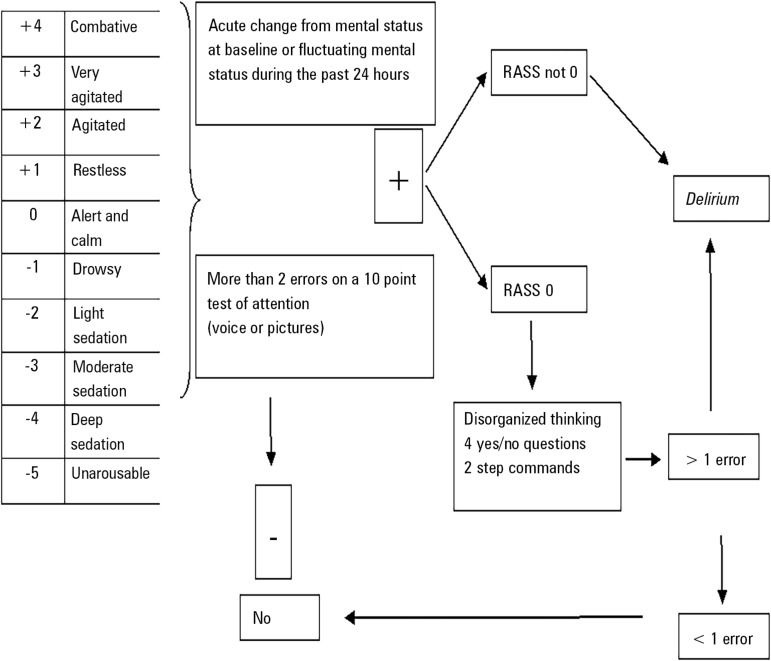
The *delirium* diagnosis flow chart according to the *Richmond Agitation-Sedation Scale* and *Confusion Assessment Method for the Intensive Care Unit* is shown. Any RASS score of -4 or -5 was considered comatose and precluded *delirium* evaluation. Change in mental status was assessed by the patient's family or nurses. RASS - Richmond Agitation Sedation Scale; CAM-ICU - Confusion Assessment Method for the Intensive Care Unit.

Figure 1 shows the diagnostic flow chart used in our investigation. The first step was to assess consciousness status using the RASS. If the patient was alert, the status was an RASS score equal to or greater than - 3, following which the CAM-ICU was performed. Accordingly, an acute change in mental status from baseline or a fluctuating mental status during the previous 24 hours as well as inattention were then evaluated. If these parameters were altered and the patient had an RASS score different from 0, a *delirium* diagnosis was confirmed (as per the CAM-ICU algorithm - http://www.icudelirium.org/docs/CAM_ICU_flowsheet_Spanish.pdf).

The RASS was evaluated in the morning before CAM-ICU data were collected and during periods of hyperactivity as well as when there were agitation periods registered by the nursing staff.

The CAM-ICU was performed daily in the morning shift and, in cases of agitation, during other shifts.

*Delirium* was categorized as follows: Hyperactive patients were CAM-ICU positive and agitated, restless or emotionally labile (*i.e*., RASS +1 to +4). Hypoactive *delirium* was characterized when patients were CAM-ICU positive and the RASS was between 0 and -3. Such patients had decreased responsiveness, apathy, lethargy, reduced motor activity, incoherent speech and lack of interest in interactions due to their inattention. If the patient presented a combination of both hyperactive and hypoactive delirium, it was considered a mixed *delirium*. Hallucinations or delusions are common in both forms but are not diagnostic criteria for *delirium*.

Any positivity noted within a 24-hour period was considered a "positive" *delirium* day.

Prospectively, we collected information on age, sex, diagnosis, medical history (alcohol intake, illicit drugs abuse, smoke, non-severe neurological psychiatric diseases), duration in the ICU and in-hospital LOS, Acute Physiology and Chronic Health Evaluation II (APACHE II) scores, total MV days, MV days prior to the first episode of *delirium*, RASS, Glasgow Coma Scale, CAM-ICU, sedative and analgesic drugs and number of days of use, use of neuroleptic drugs and intravenous steroid use.

Patients were also categorized according to the ICU admission diagnosis: (1) medical or surgical patients and (2) according to the ICU's admission diagnostic codes, which include 27 diseases.

RASS and CAM-ICU monitoring were performed while patients were under invasive mechanical ventilation.

All study variables, including outcome (death or recovery), were recorded until ICU discharge.

At hospital discharge, only the outcome and length of stay were registered.

### Statistical analysis

Demographic and clinical variables were summarized using descriptive statistics. Continuous variables were described using the mean and standard deviation (SD) or median and interquartile range, depending on data distribution. Normal distribution was assessed using the Kolmogórov-Smirnov test. A comparison between *delirium* and non-*delirium* groups for continuous variables was performed using Student's *t*-test for independent samples with normal distribution, the Mann-Whitney U test for samples in which the distribution was not normal, and the chi-square test or Fischer's exact test for qualitative variables. P-values less than 0.05 were considered significant.

To establish risk factors associated with *delirium* development and death at ICU discharge, a logistic regression analysis was performed.

Variables that showed p < 0.20 in the univariate analysis and those considered clinically relevant were elected to compose the multivariate analysis. The results of the multivariate analysis are expressed as odds ratios with 95% confidence intervals.

State Software (State Corp LP, version 13) was used for all calculations.

## RESULTS

**Figure 2:**
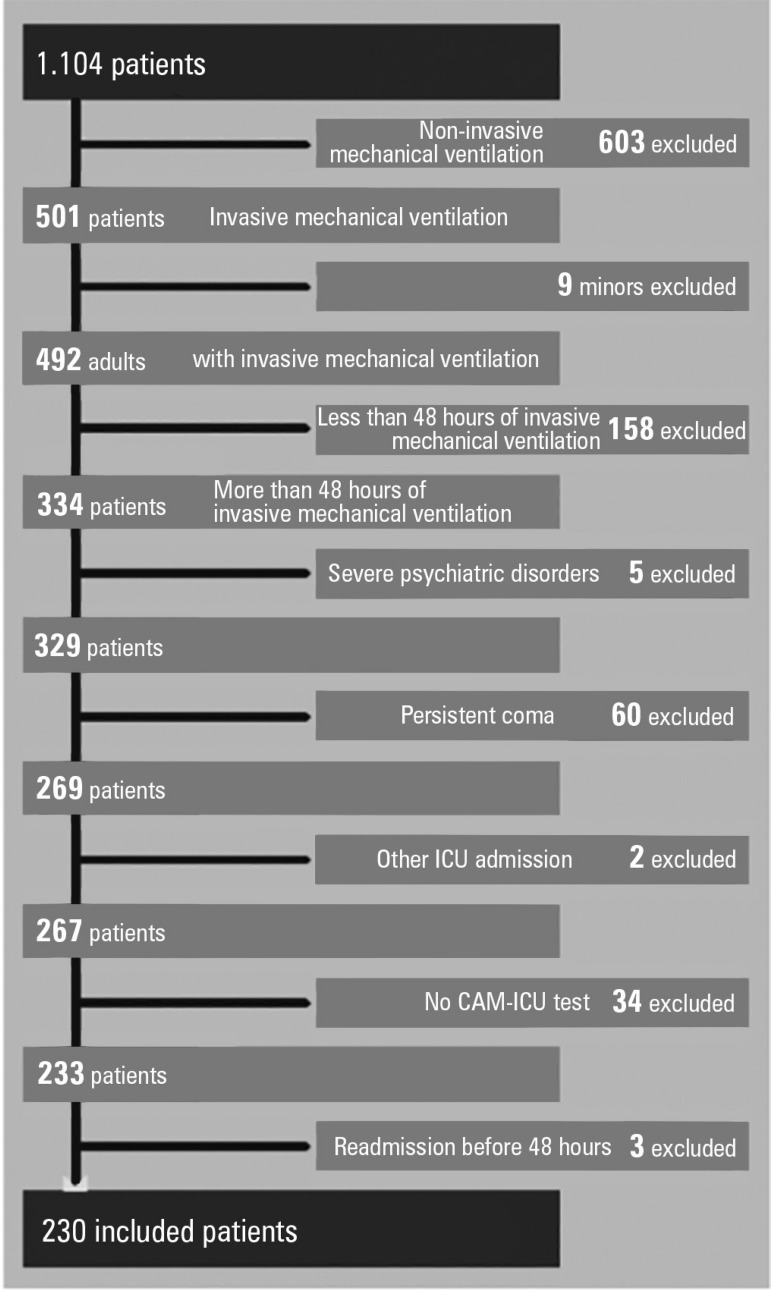
Flow chart. ICU - intensive care unit; CAM-ICU - Confusion Assessment Method for the Intensive Care Unit.

From April 20, 2014 to April 20, 2015, 1104 patients were admitted to the ICU. From these patients, 290 fulfilled the inclusion criteria, and 230 patients were included in the study (Figure 2). In table 1, the cohort-baseline characteristics are shown for all patients according to *delirium* status (never *delirium versus* ever *delirium*).

**Table 1:** Baseline characteristics and clinical outcomes by delirium status

Variable	All patients (N = 230)	No delirium (N = 46)	Delirium (N = 184)	p value
Age	60.6 (17.7)	55.91 (17.88)	61.77 (17.57)	0.04*
> 65	115 (50.0)	19 (41.3)	104 (56.5)	0.06†
Sex				
Male	140 (60.9)	23 (50.0)	117 (63.6)	0.09†
Medical history				
Alcohol consumption	64 (28.6)	7 (16.3)	57 (31.5)	0.04†
Tobacco use	116 (53.7)	19 (45.2)	97 (55.7)	0.2†
Drug abuse	24 (10.4)	6 (13)	18 (9.8)	0.5†
HIV	5 (2.2)	0 (0)	5 (2.7)	0.25†
Psychiatric disorder	43 (18.7)	5 (10.9)	38 (20.7)	0.13†
Stroke	29 (12.6)	5 (10.9)	24 (13)	0.6†
Disease severity				
APACHE II score	24.21 (9.07)	22.39 (8.38)	24.66 (9.20)	0.17*
Disease stratification				
Medical	172 (74.8)	34 (73.9)	138 (75)	0.8†
Surgical	88 (25.2)	12 (26.1)	46 (25)	
In-hospital length of stay	21.11 (19.11)	17.26 (15.65)	22.56 (20.84)	0.01‡
Mechanical ventilation (days)	6 (3 - 11)	3.5	7	0.001‡
Analgesia (days)	2 (1 - 3)	1	2	0.001‡
Sedation (days)	2 (1 - 3)	1	2	0.0001‡
Mortality				
In ICU	46 (20)	5 (10.9)	41 (22.3)	0.08†
In hospital	106 (46)	15 (32)	95 (51)	0.01†

HIV - human immunodeficiency virus; APACHE II - Acute Physiology and Chronic Health Evaluation; ICU - intensive care unit. Values expressed as the N (%), means (standard deviation), or medians and interquartile range.

* Student’s *t*-test for independent samples;

† Chi square;

‡ Mann-Whitney U test for independent samples.

The prevalence of *delirium* was 80% (N = 184); the mean onset was at 3.6 days (SD 3.7), and the mean duration was 4 days (SD 2 - 6.75). The number of patients according to *delirium* psychomotor subtypes was as follows: 11 hyperactive patients (6%), 9 hypoactive patients (5%) and 160 mixed patients (89%).

We found significant differences between the *delirium* group and the non-*delirium* group regarding analgesia and sedation days that were noted in the clinical outcomes including ICU LOS (p = 0.01) and hospital LOS (p = 0.01) as well as hospital mortality (p = 0.01) (Table 1).

Two logistic regression models were performed with the first one using *delirium* as a dependent variable and the second one using mortality as a dependent variable.

For the first model, using *delirium* as a dependent variable, the variables included were as follows: age over 65 years, a history of alcohol consumption, psychiatric disorders, post-surgical pathology, gender and number of mechanical ventilation days prior to the first episode of *delirium* (Table 2). Multivariable analysis revealed that age over 65 years, a history of alcohol consumption, surgical pathology and number of mechanical ventilation days prior to the first episode of *delirium* were independent variables associated with *delirium* development (Table 2).

**Table 2:** Multiple logistic regression model using delirium as the dependent variable

Variables	Multivariate analysis		
	OR	p value	95%CI
Age > 65	1.02	0.03	1.003 - 1.048
Alcohol consumption	2.90	0.03	1.11 - 7.89
Surgical patients	0.26	0.04	0.07 - 0.96
Days of ventilation prior to *delirium* onset (ref: 0-1 day)	1	0.001	
2	0.09	0.001	0.02 - 0.38
3	0.18	0.024	0.04 - 0.79
4+	0.17	0.008	0.04 - 0.63

OR - odds ratio; 95%CI - 95% confidence interval. Included only variables with p < 0.2.

For the second model, using ICU mortality as a dependent variable, the variables included were as follows: *delirium*, number of analgesia days, sedation days and MV days, use of intravenous steroids, APACHE II scores, acute coronary syndrome, shock and sepsis. Multivariable analysis revealed that APACHE II values, MV for more than 7 days, acute coronary syndrome and number of sedation days were independent variables associated with ICU mortality (Table 3).

**Table 3:** Multiple logistic regression model using intensive care unit mortality as the outcome variable

Variables	Multivariate		
	OR	p value	95%CI
Sedation days	1.26	0.008	1.07 - 1.48
Total mechanical ventilation > 7 days	3.58	0.001	1.59 - 8.08
APACHE II	1.07	0.029	1.02 - 1.13
Acute coronary syndrome	11.79	0.029	1.34 - 103.2

OR - odds ratio; 95%CI - 95% confidence interval; APACHE II - Acute Physiology and Chronic Health Evaluation II. Included only variables with p < 0.2.

## DISCUSSION

We prospectively evaluated incidence, outcomes, and predictors of *delirium* in mechanically ventilated critically ill patients in a general ICU (medical, coronary, surgical and trauma) using CAM-ICU as a diagnostic tool. In this cohort, the prevalence of *delirium* was 80% (N = 184). Older age, alcohol consumption, extended in-hospital LOS, MV for more than 7 days, use of analgesia and sedation and in-hospital mortality were associated with *delirium* development. The multivariate regression model using *delirium* as a dependent variable identified age > 65 years, a history of alcohol consumption, perioperative period and MV for more than 3 days as independent variables.

The other multivariate regression model using ICU mortality as a dependent variable recognized number of sedation days, mechanical ventilation for more than 7 days, APACHE II scores and acute coronary syndrome as independent variables.

*Delirium* prevalence was commensurate with previous literature.^(2,14,15)^ According to Salluh's meta-analysis of 44 articles (N = 16,595 patients), only 8 papers revealed a prevalence of *delirium* greater than 60%.^(1)^

In one paper, Cruz et al.,^(16)^ stated that there is a broad range of *delirium* incidence that may change according to the type of studied patients, the interpretation of clinical findings and the diagnostic screening tools.

If confirmed in other regional ICUs, this high *delirium* rate is a clarion call for quality improvement efforts in Latin America designed to reduce the burden of acute brain dysfunction in these ICU patients.

Furthermore, this cohort identified that the duration of mechanical ventilation was the most consistent independent predictor of both *delirium* and high mortality. This provides a target for future work such as the implementation of the ABCDEF bundle and the ICU Liberation Collaborative, since these evidence-based approaches have been shown to improve outcomes including a reduction in duration of mechanical ventilation and *delirium* and mortality.^(17-29)^

Several cohort studies^(1,28)^ have shown that in six-month and one-year periods, the risk of mortality increased by 10% per day that an ICU patient was delirious. Moreover, it is known that the duration of *delirium* is an independent predictor of cognitive impairment.

Mehta et al.^(30)^ reported 3.6 days for *delirium* onset and a duration of 2 days, while Klein Klouwenberg et al.^(31)^ reported a duration of 3 days and Shehabi^(15)^ reported a duration of 4 days. The latter author found a dose-response increase in mortality with increasing durations of *delirium* from 0 to 1, 2, and 3 *delirium* days. We used a similar interval from 1 to 3 days and more than 4 days and arrived at consistent findings.

*Delirium* psychomotor behavior was categorized as hyperactive in 11 patients (6%), hypoactive in 9 patients (5%) and mixed in 160 patients (89%). In Peterson's study,^(32)^ similar findings occurred including a mixed type, which was the most common type (54.9%). Thus, most of the *delirium* observed in the ICU is hypoactive or mixed, which requires routine monitoring for detection given that 75% of hypoactive *delirium* is missed.^(33-35)^

Hyperactivity and agitation occur frequently in ICU patients. Almeida et al.^(33)^ found that *delirium* was an independent risk factor for agitation in the first 7 days after ICU admission.

Risk factors for *delirium* could be predisposing or precipitating risk factors. Older age is a well-known predisposing factor widely recognized and closely related to other predisposing factors such as multiple comorbidities including underlying cognitive impairment. Our finding that age over 65 years is an independent *delirium* factor is in accordance with these previous data. McNicoll et al.^(36)^ found four preexisting factors for *delirium* in older patients admitted to medical ICUs (dementia, administration of benzodiazepines before ICU admission, elevated creatinine levels and low arterial pH). In Uruguay, the elderly population is growing; consequently, there is an increase in admission rate for elderly patients to the ICU. Due to this aging population, the number of patients at risk of *delirium* and neuropsychiatric disorders is large and should serve to initiate all the quality improvement that is possible.

As explained in Pitrowsky's^(37)^ study, alcohol consumption and gender are two non-modifiable factors. In our study, both male gender and alcohol consumption were significant for the occurrence of *delirium* according to the univariate analysis. Alcohol is associated with *delirium*, which agrees with most of the findings included in the literature that identify alcohol as a predisposing risk factor for *delirium*. Male gender was associated with alcoholism in our univariate analysis.

The development of *delirium* in the ICU was an important predictor of increased mortality,^(2)^ and clinical practice guidelines conclude that *delirium* is associated with high mortality in adult ICU patients. Guidelines also report with high quality evidence that *delirium* is associated with prolonged ICU and hospital LOS in adult ICU patients. Our results agree with these findings. Regarding other outcomes, our data showed that *delirium* was associated with significantly increased ICU and hospital LOS. In Salluh's^(1)^ meta-analysis, 28 studies reported that ICU LOS was significantly longer in patients with *delirium*. Regarding hospital LOS, 22 studies reported that the LOS was significantly longer in patients with *delirium*.

In Salluh's paper,^(1)^ 10 studies reported that MV duration was a predictor of *delirium* development. In our series, the number of MV days was 3.5 days for the non-*delirium* group and 7 days for the *delirium* patients, which was very close to the findings by van den Boogaard et al.^(38)^ but dissimilar from the average difference of 1.79 days in Salluh's^(1)^ meta-analysis. The logistic regression model using either *delirium* or mortality as dependent variables exhibited number of MV days as independent predictors of the unwanted outcome. However, it is true that more MV days might merely indicate sicker patients, and the higher mortality rate might intuitively reflect this higher severity of illness. However, numerous other investigators have adjusted for severity of illness and found that *delirium* is an independent predictor of mortality, which was similar to our findings. Thus, these data complement prior work and for only the second time, provide data from Latin America about the association between *delirium* and mechanical ventilation and mortality.

We did not find an association between analgesia and sedation days and *delirium* onset. Multiple publications have shown that benzodiazepines are associated with higher rates of *delirium*^(39,40)^ and that by reducing benzodiazepines, *delirium* prevalence is decreased.^(16-20,22)^ Although there were significant differences between delirious and non-delirious patients, regarding the number of analgesia and sedation days prior to the start of *delirium*, multiple logistic regression models using *delirium* as the dependent variable revealed no significant relationship. It is noteworthy that our ICU policy on analgesia and sedation is restrictive; consequently, the difference in the average number of sedation days (1 day *versus* 2 days) is far less than that reported in the literature. Previous randomized trials that have compared daily sedation interruption with control strategies have helped form a basis for the current ABCDEF Care Bundle and the ICU Liberation Collaborative.^(41)^ It is clear that different approaches towards sedation reduction are possible, and these should be embraced at the local level to increase compliance and overall success. In the end, however, this is likely a key element for future clinical attempts and investigations to reduce mechanical ventilation duration in Latin America with the overarching goal to improve the epidemiology of *delirium* and hospital mortality.

This investigation has important limitations that must be addressed. First, the ICU in this study was present in one region of a major city in Uruguay, which is strong methodologically, yet these data may not be generalizable to other regions of Latin America. Second, the modeling used was a univariate/multivariate approach rather than an *a priori* establishment of the clinically relevant covariates, which is preferred by many biostatisticians. In addition, many variables were handled as dichotomous thresholds (e.g., *delirium* ever/never, age > 65) rather than as continuous or time-varying covariates. Even considering this limitation, however, the results were very consistent with prior work and thus carry face validity. Some variables did not reach significance due to a small sample size. It is well known that studies with a small number of participants are subject to bias, even if the number to reach statistical significance in the sample size calculation is adequate.

The main findings of this cohort will likely be used to initiate better quality improvement in Latin American ICUs where many clinicians have been perhaps overly satisfied with the status quo. Having had very few data from the entire continent other than from Brazil, it has been easy to assume that no problem existed; however, these 80% *delirium* rates cannot be ignored. One of our study strengths was the health team training in RASS and CAM-ICU since these tools were not routinely used before the protocol implementation. Routine use of RASS and CAM-ICU, which is recommended as usual care (but poorly implemented) in the Pain, Agitation, and *Delirium* Guidelines, allowed us to identify that 80% of these ventilated Uruguayan ICU patients suffered from *delirium*, which is consistent with previous cohorts but certainly higher than preferred. The findings are complementary to those of other regions of the world and represent only the second prospective dataset from Latin America in this field.

Lastly, future work should expand the size and regions studied as well as utilize interventions and long-term cognitive and physical outcomes, neither of which was measured in this investigation.

## CONCLUSION

This prospective cohort investigation provides novel data from Latin America to further our understanding of the epidemiology of *delirium* and mortality in intensive care unit patients in this region. Routine use of the RASS and CAM-ICU allowed us to identify an 80% delirium rate in which risk factors for *delirium* development were age older than 65 years, alcohol consumption, perioperative period and number of mechanical ventilation days. *Delirium* was associated with extensive intensive care unit and hospital length of stay; and in-hospital mortality.

Hospital mortality risk factors were number of sedation days, illness severity graded as APACHE II measurements and the presence of acute coronary syndrome.
