# ERRATUM

**DOI:** 10.5935/0103-507X.20170059

**Published:** 2017

In the manuscript ***Delirium* in a Latin American intensive care unit. A prospective cohort study of mechanically ventilated patients**, DOI number 10.5935/0103-507X.20170058, published at Rev Bras Ter Intensiva. 2017;29(3):337-45, there was a spelling error in one of the authors’ surname. The author’s name was corrected from: **Ely Wesley** to: **E Wesley Ely**.

